# Bilateral Sensorimotor Impairments in Individuals with Unilateral Chronic Ankle Instability: A Systematic Review and Meta-Analysis

**DOI:** 10.1186/s40798-024-00702-y

**Published:** 2024-04-08

**Authors:** Xiaomei Hu, Tianyi Feng, Pan Li, Jingjing Liao, Lin Wang

**Affiliations:** Key Laboratory of Exercise and Health Sciences, Shanghai University of Sport, Ministry of Education, Shanghai, China

**Keywords:** Bilateral deficits, Sensorimotor, Neuromuscular control, Balance, Proprioception, Kinaesthesia, Injured, Uninjured

## Abstract

**Background:**

Chronic ankle instability (CAI) is manifested by sensorimotor impairments in the sprained ankle, including deficits in sensation, motor function, and central integration or processing. These impairments have a significant impact on physical activities and daily life. Recently, some studies have suggested that bilateral deficits were observed in unilateral CAI, but contradictory evidence disputes this finding. Therefore, the objective of this study was to investigate whether bilateral sensorimotor deficits presented in individuals with unilateral CAI.

**Methods:**

Without language restriction, the following databases were retrieved from database inception up until 3 November 2023, including PubMed, WOS, EMBASE, Cochrane, SPORTDiscus and CINAHL. Case-control and cross-sectional studies that investigated bilateral sensorimotor functions in individuals with unilateral CAI were included. Sensorimotor functions contained static and dynamic balance, functional performance, muscle strength and activation, as well as sensation. Outcome measures contained centre-of-pressure parameters, normalised reach distance, activation time and magnitude of muscle, sensory errors and threshold. The risk of bias and quality assessment of included studies were evaluated using a standardised tool recommended by the Cochrane Collaboration and the Epidemiological Appraisal Instrument, respectively. To explore the potential bilateral deficits associated with unilateral CAI, a comprehensive meta-analysis was conducted using Review Manager version 5.4. The analysis compared the injured limb of unilateral CAI with healthy controls and the uninjured limb with healthy controls. The main focus of this study was to investigate the differences between the uninjured limb and healthy controls. A random-effects model was employed and effect sizes were estimated using the standardised mean difference (SMD) with 95% confidence intervals (CIs). Effect sizes were deemed as weak (0.2–0.5), moderate (0.5–0.8), or large (> 0.8).

**Results:**

A total of 11,442 studies were found; 30 studies were contained in the systematic review and 20 studies were included in the meta-analysis. Compared with healthy controls, those with unilateral CAI presented weak to moderate impairments in their uninjured limbs in static balance with eyes open (SMD = 0.32, 95% CI: 0.08 to 0.56), functional performance (SMD = 0.37; 95% CI: 0.08 to 0.67), kinesthesia (SMD = 0.52; 95% CI: 0.09 to 0.95) and tibialis anterior activation (SMD = 0.60, 95% CI: 0.19 to 1.01). There were no significant differences in other comparisons between the uninjured limb and healthy controls.

**Conclusions:**

Patients with unilateral CAI may present bilateral deficits in static balance with eyes open, functional performance and kinaesthesia. However, further evidence is required to confirm this point due to limited studies included in some analyses and small effect size.

**Registration:**

The protocol was registered in the International Prospective Register of Systematic Reviews platform (CRD: 42,022,375,855).

**Supplementary Information:**

The online version contains supplementary material available at 10.1186/s40798-024-00702-y.

## Abstract (key points)

**Key Points**

1. This study conducted a meta-analysis to investigate bilateral functions in patients with unilateral chronic ankle instability (CAI). The analysis compared the injured limb of unilateral CAI with healthy control and the uninjured limb with healthy control.
2. Patients with unilateral CAI may present bilateral deficits in static balance with eyes open, functional performance and kinesthesia. However, further large-scale studies are required to validate these findings.
3. In future research, it is crucial to apply bilateral assessment in individuals with unilateral CAI and explore interventions targeting both limbs. Additionally, considering the differences between mechanical and functional ankle instability, future studies could explore the bilateral characteristics based on this classification to gain a better understanding of the variations between the two. This will provide more specific guidance for developing effective training methods.

**Supplementary Information:**

The online version contains supplementary material available at 10.1186/s40798-024-00702-y.

## Background

Ankle sprains are common sport-related injuries with a high incidence and recurrence rate, among which lateral ankle sprains are the most common, accounting for approximately 80–90% of ankle sprains [1, 2]. However, up to 70% of people who experience acute lateral ankle sprains continue to suffer from residual symptoms, including recurrent ankle sprains, persistent pain, swelling and self-reported instability, while around 40% of individuals develop chronic ankle instability (CAI) [2, 3]. Compared with healthy individuals, patients with CAI present lower levels of physical activity participation, decreased quality of life and increased risk of developing post-traumatic osteoarthritis [1, 2]. Furthermore, CAI imposes substantial healthcare costs for management and treatment [1, 2, 4]. Therefore, it is essential to investigate the factors contributing to CAI and the functional limitations associated with CAI to develop related interventions and management approaches.

The sensorimotor system, which plays a crucial role in maintaining postural stability, encompasses various sensory, motor and central integration components involved in preserving joint homeostasis during bodily movements [5]. Assessing sensorimotor function involves multiple methods such as balance tests, functional performance evaluations, muscle strength and activation assessments and sensation assessments [4, 6, 7]. Extensive evidence has demonstrated that CAI is associated with sensorimotor impairments in the injured ankle, contributing to postural instability in patients with CAI [4, 8–12]. However, an interesting but paradigm-challenging phenomenon observed in recent studies targeting bilateral assessment of unilateral CAI is that deficits in unilateral CAI could be observed bilaterally [13–15]. This indicates that increased sensorimotor impairments present not only in the injured limb but also in the uninjured limb, which means alterations in the injured limb of unilateral CAI may influence the functions of the contralateral side [16, 17]. These bilateral observations regarding unilateral CAI suggest that there may be a centrally mediated process involved in the development of unilateral CAI, which contributes to bilateral impairments. However, there is no definitive evidence in the existing literature.

Sprains may damage the peripheral mechanoreceptors, which are essential for perceiving and modulating posture, resulting in disrupted sensory afferents on the injured side [16–18]. In CAI, the sensitivity of the muscle spindles, one of the main mechanoreceptors, can be impaired [13]. It has been shown that articular and cutaneous afferents, together with muscle afferents and descending supraspinal commands, activate the γ motoneuron pool [5]. The decreased sensory afferents in the ankle could lead to suppressed γ motoneuron activation and subsequent incorrect motor output [5]. These changes may influence the processing and integration of the central nervous system, leading to reorganization or adaptive alterations [19]. Due to the complex interactions between the two hemispheres, one hemisphere may influence sensorimotor functions in the bilateral hemisphere [18]. The bilateral deficits have been observed in acute lateral ankle sprain, which provides further evidence for centrally mediated changes [16, 17, 20]. CAI develops from an acute lateral ankle sprain, and it may also present bilateral changes. However, the evidence for bilateral deficits in CAI is insufficient and contradictory. Therefore, evidence needs to be pooled quantitatively to clarify this observation and investigate possible mechanisms for bilateral changes to guide further research.

Currently, the majority of assessments and interventions in individuals with unilateral CAI primarily concentrate on the injured side. Considering the potential bilateral sensorimotor impairments, understanding the bilateral alterations could contribute to clinical assessments, treatments and scientific research endeavors. Accordingly, the primary aim of this study was to determine whether patients with unilateral CAI present bilateral deficits in comparison to healthy individuals. We hypothesised that patients with unilateral CAI would exhibit bilateral deficits.

## Methods

This systematic review and meta-analysis was conducted in accordance with the PRISMA checklist and registered in the International Prospective Register of Systematic Reviews platform on November 25, 2022 (CRD: 42,022,375,855).

### Search Strategy

Two authors (XH and TF) individually conducted a literature retrieval in six electronic databases, namely, PubMed, WOS, EMBASE, Cochrane, SPORTDiscus and CINAHL, from the inception of each database up to 3 November 2023, without language restriction. MeSH terms or keywords were used during the literature search. The following keywords were used in PubMed: (‘bilateral’ OR ‘unilateral’ OR ‘contralateral’ OR ‘ipsilateral’ OR ‘side to side’ OR ‘side-to-side’ OR ‘injured’ OR ‘uninjured’ OR ‘affected’ OR ‘unaffected’ OR ‘impair*’ OR ‘unimpaired’ OR ‘dominant’ OR ‘non-dominant’) AND (‘ankle instability’ OR ‘unstable ankle’ OR ‘ankle sprain*’ OR ‘ankle injur*’). Additionally, the reference lists of full-text papers also were manually searched to identify studies that were not retrieved through the database search.

### Study Selection and Data Extraction

We included cross-sectional and case-control studies that investigated bilateral sensorimotor function in those who unilateral CAI compared with healthy controls [1]. To be diagnosed with CAI, individuals should exhibit clinical symptoms, which include experiencing at least one severe ankle sprain, recurring ankle sprains and self-reported instability. Eligible studies focused on functional ankle instability (FAI), which is characterized by deficiencies in proprioception, neuromuscular control, strength and postural control, or mechanical ankle instability (MAI) characterized by pathologic laxity, arthrokinematic restrictions, degenerative changes and synovial changes. Studies that were excluded from the analysis included those involving patients with bilateral CAI, those solely comparing the injured and uninjured sides of CAI, or those lacking a healthy control group. In addition, reviews, case studies, animal studies and not fully peer-reviewed papers were removed. For non-Chinese and non-English studies, DeepL translator was used for translation [21].

The testing protocol had to include at least one of the following components: static or dynamic balance test, functional performance test, muscle strength or activation assessment and sensory evaluation. Static balance is assessed by the single-leg stance test, with outcomes measured through parameters such as center of pressure (COP), error count, time-to-boundary analysis or stability index. Dynamic balance is measured by tests such as the Star Excursion Balance Test (SEBT), instruments (e.g., Biodex Balance System), walking test, jump-landing task and other relevant measures. Outcome measures for these assessments should encompass variables such as normalised reach direction, stability index, time to stability and COP-based parameters. Functional performance encompasses various hops, including figure-of-8 hop, side hop and square hop among others, and the outcome measure is typically the time taken to complete the assigned testing task. Assessments of muscle strength involve measurements of isokinetic strength and maximal voluntary isometric contractions, with the average peak torque serving as the primary outcome measure. In terms of muscle activation, techniques such as surface electromyography (sEMG) and EMG are commonly employed. Mean amplitudes, root mean squares, activation magnitude, delayed activation time and other related variables can be used as outcomes. Regarding sensory assessment, studies should cover at least one kind of sensory evaluation, including somatosensation, vision and vestibular. Somatosensation includes proprioception, tactile, pain and temperature perception. Proprioception specifically refers to joint position sense, force sense and kinesthesia. Outcome measures for sensations could include variables, including joint position replication error, force sense replication error and sensory threshold.

After removing the duplicates, the search results were independently assessed by two authors (XH and TF) based on titles, abstracts and full texts. Any discrepancies were handled by consulting the third author (PL). A Microsoft Excel (2019) table was applied to extract relevant information including study design, population, sample size, details of test methodology and related outcomes. In cases where numerical data were unclear or unreported, contact was made with the authors via email to request clarification. For data reported graphically, Engauge Digitizer 4.1 was used to extract the values from the graph [22].

### Quality and Risk of Bias Assessment

The Epidemiological Appraisal Instrument (EAI), a tool with good validity and reliability, was applied to estimate the quality of included studies [4, 6, 10, 23]. The original EAI contains 43 items, each scored as ‘Yes’ (1 score), ‘Partial’ (0.5 score), ‘no’ or ‘unable to determine’ (0 score) and ‘not applicable’ [23]. Ten items from the EAI were deemed ‘not applicable’ across all studies and excluded due to the case-control and cross-sectional designs [4]. Consequently, 33 items were used to assess the observational studies in the current study [4, 6, 10]. Finally, the average score (0–1) was calculated as the overall quality of each included study except for the items scored as ‘not applicable’ [4]. The overall quality for each included study was derived by summing all responses (e.g. yes, partial, no/unable to determine) and dividing by the 33 items (score 0–1) [4].

The risk of bias was assessed using a standardised tool recommended by the Cochrane Collaboration for the review of non-randomised studies [4, 6, 10, 24]. This tool contains five domains: selection, performance, detection, attrition and reporting bias [24].

Moreover, the recommendations provided by the International Ankle Consortium were used to estimate the variability of individuals with CAI [4, 6, 10, 25]. The standard inclusion criteria for CAI include the following requirements: (1) a history of at least one significant ankle sprain occurring ≥ 12 months ago, resulting in pain, swelling and physical activity constraints for at least one day, (2) no ankle sprain within the last 3 months, (3) presence of at least one of the three classical symptoms of CAI, including experiencing a minimum of two episodes of ‘giving way’ within the past 6 months, encountering at least two ankle sprains on the same ankle, and self-reported instability evaluated via reliable questionnaires [25]. Each item was scored as ‘Reported’ (1 point), ‘Partial’ (0.5 point) and ‘Not reported’ (0 point) [10].

Before rating, all reviewers thoroughly examined the specifics of each item and reached an agreement. Afterwards, two authors (XH and PL) independently rated the included studies, and any disagreements were resolved by consulting the third reviewer (JL).

### Statistical Analysis

The sensorimotor deficits in unilateral CAI were investigated through meta-analyses using a random-effects model in Review Manager version 5.4. Pooled standardised mean difference (SMD) and corresponding 95% confidence intervals (CIs) were calculated. Subgroup analyses were conducted when appropriate. The magnitude of the effect size (ES) was determined by the SMD value, with values ranging from 0.2 to 0.5 indicating a weak effect, 0.5 to 0.8 representing moderate, and > 0.8 representing a large ES [26]. In addition, *Q* and *I*^*2*^ statistics were calculated to determine the heterogeneity, in which *p* < 0.05 was deemed significant, and *I*^*2*^ ≥ 75% represents high heterogeneity, indicating the consequences should be interpreted cautiously [4, 6, 27]. Publication bias was assessed using a funnel plot. The robustness of the pooled results was assessed by conducting sensitivity analyses by removing the studies with large effects [4, 10].

The kappa value was applied to assess the inter-rater consistency between the two reviewers, and this value was calculated using SPSS version 25.0 (IBM Corp., Armonk, NY, USA). The inter-rater agreements were deemed as poor (< 0.00), slight (0–0.2), fair (0.21–0.4), moderate (0.41–0.6), substantial (0.61–0.8) and almost perfect (0.81–1.0) [28].

## Results

### Study Selection and Characteristics

**Fig. 1:**
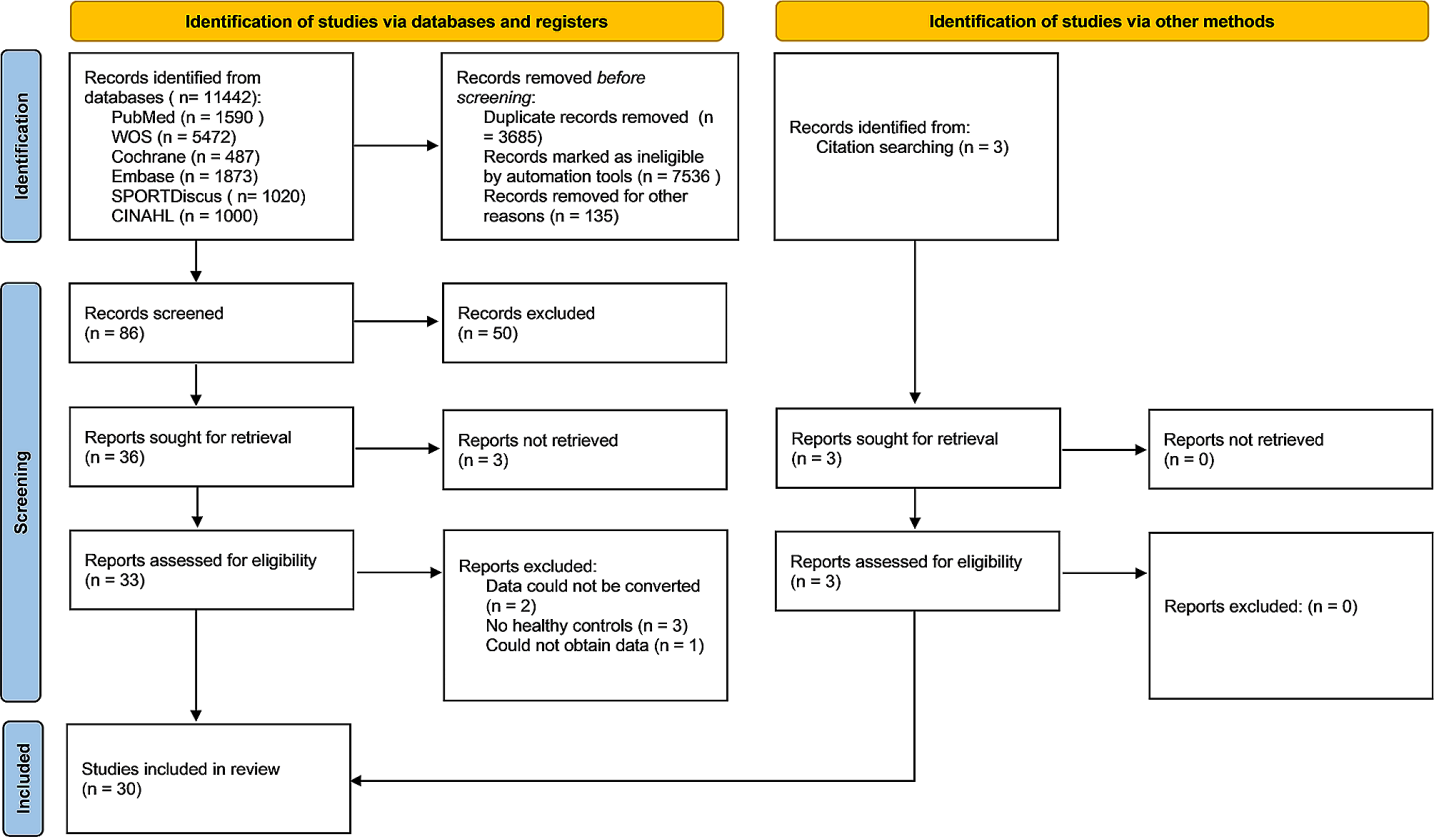
Flow chart of the study selection process

In total, 11,442 potential papers were identified via systematic search. After removing 3,685 duplicates and screening titles and abstracts, 86 studies met the eligibility criteria, with 20 studies included in the final meta-analysis. The flowchart (Fig. 1) shows the whole selection process and reasons for exclusion. From the included studies, 17 papers investigated postural control, including 6 studies on static balance [29–34], 11 studies on dynamic balance [32, 35–41] and 3 studies on functional performance [7, 39, 42]. Seven studies focused on muscle strength and activation [32, 43–48], while only two studies [13, 34] explored proprioception in patients with unilateral CAI. Separate meta-analyses were conducted for static and dynamic balance, functional performance, ankle muscle strength and activation and ankle proprioception to evaluate bilateral sensorimotor function in patients with CAI compared with healthy individuals. The average age of individuals with CAI ranged from 17.1 to 41.0 years across the 20 included studies, while the age range of healthy controls varied from 14.6 to 42.0 years. Notably, Hiller et al. [31] included participants below the age of 18 years, and Santos and Liu [34] encompassed a wide age range. The types of CAI included both FAI and MAI as previously reported. Further details regarding eligible studies, including study design, sex, sample size, outcome measure methodologies, measurement devices and related outcomes, are provided in Supplementary Material 1. An attempt was made to obtain unreported numerical data by contacting one author via email. Unfortunately, no response was received.

### Quality and Risk of Bias Assessment

For the quality assessment of included studies, the agreement between two authors was almost perfect (*k* = 0.917, *p* < 0.001) with 625 agreements from 660 items, and the rating scores presented the median 0.52 (range 0.44–0.62). The results of the quality evaluation indicated that each study had well-defined objectives, comprehensive reporting of outcomes, clear eligibility criteria, appropriate statistical techniques and valid assessment procedures. However, certain areas displayed deficiencies across several studies. Specifically, only three studies provided sample size calculations, while blinding was implemented in five studies. Furthermore, none of the studies provided results categorized by CAI severity and only six studies reported or controlled for confounding factors. The full EAI results are provided in Supplementary Material 2.

The risk-of-bias evaluation can be found in Supplementary Material 3, and 133 agreements were achieved from 140 items with almost perfect inter-rater agreement (*k* = 0.809, *p* < 0.001). Almost all studies described the demographic information, used the appropriate testing devices and conducted relevant comparisons. However, a high risk of bias was observed in terms of blinding and comprehensive descriptions of participant characteristics. Besides, funnel plots indicated the presence of publication bias mainly in the analysis of static balance, dynamic balance and functional performance when comparing injured limb with control.

When assessing the variability among individuals with CAI, 135 agreements were reached from 140 items with almost perfect inter-rater agreement (*k* = 0.945, *p* < 0.001). Regarding the criteria recommended by the International Ankle Consortium, all studies required participants to have experienced at least one significant ankle sprain. However, only 45% of the studies reported that the sprain resulted in pain, swelling, or a disruption of physical activity lasting for at least 1 day, and 55% of the studies specified that initial injury should occur at least 12 months before the study. All studies indicated that patients had not experienced an ankle sprain within the last 3 months. Additionally, 70% provided descriptions of “giving way”. A substantial proportion (55%) of the studies required participants to experience at least two ankle sprains on the same ankle. Approximately 55% of the studies included self-reported function evaluated by questionnaires, including Foot and Ankle Disability Index, Ankle Instability Instrument, Cumberland Ankle Instability Tool and Foot and Ankle Ability Measure. The details are presented in Supplementary Material 4.

### Balance and Functional Performance

**Fig. 2:**
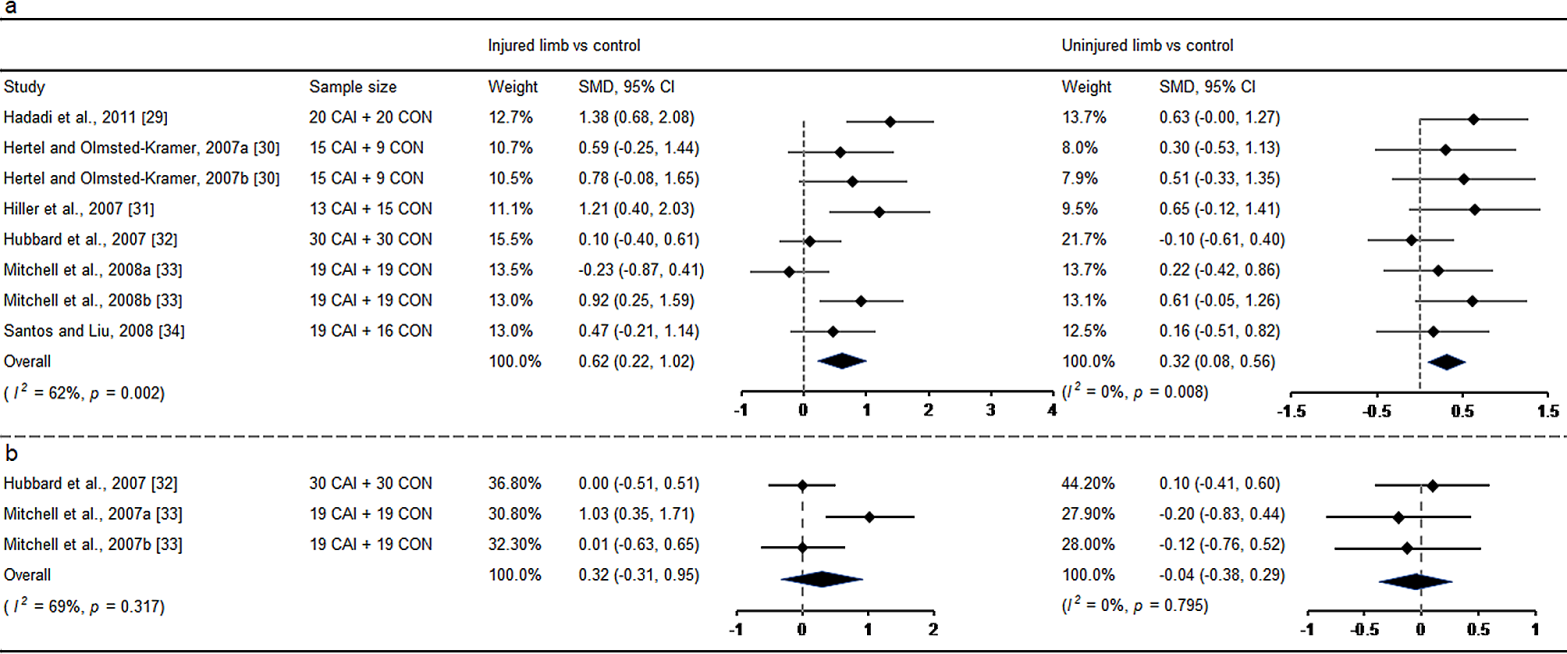
Forest plot of static balance with eyes open (**a**) and eyes closed (**b**) comparing CAI with control. Hertel and Olmest-Kramer, 2007a (velocity of centre pressure in mediolateral direction) and b (velocity of centre pressure in anteroposterior direction) [30]; Mitchell et al., 2008a (velocity of centre pressure in mediolateral direction) and b (velocity of centre pressure in anteroposterior direction) [33]. CAI chronic ankle instability, CI confidence interval, CON control, SMD standardized mean difference. Positive SMD indicates balance deficits in CAI

Seven studies [29–34, 49] investigated static balance using the single-leg stance test between individuals with CAI and healthy controls. Except for the study conducted by Hiller et al. [31], which measured the magnitude of mediolateral ankle movement during single-leg stance, all other studies [29, 30, 32–34] employed COP-based outcome measures. However, one study was not included in the meta-analysis due to the unavailability of data that could not be converted into mean and standard difference [49]. Since Hertel et al. [30] and Mitchell et al. [33] assessed the velocity of COP in mediolateral and anteroposterior directions, data from both directions were extracted. The pooled results indicated that individuals with unilateral CAI presented static balance with eyes open deficits in both the injured limb (SMD = 0.62, 95% CI: 0.22 to 1.02, *I*^*2*^ = 62%) with moderate ES and the uninjured limb (SMD = 0.32, 95% CI: 0.08 to 0.56, *I*^*2*^ = 0%) with small ES when compared with healthy controls (Fig. 2a). Two studies [32, 33] examined static balance with eyes closed also using the single-leg stance test. No significant differences were observed in both the injured and uninjured sides when compared with healthy controls (Fig. 2b).

**Fig. 3:**
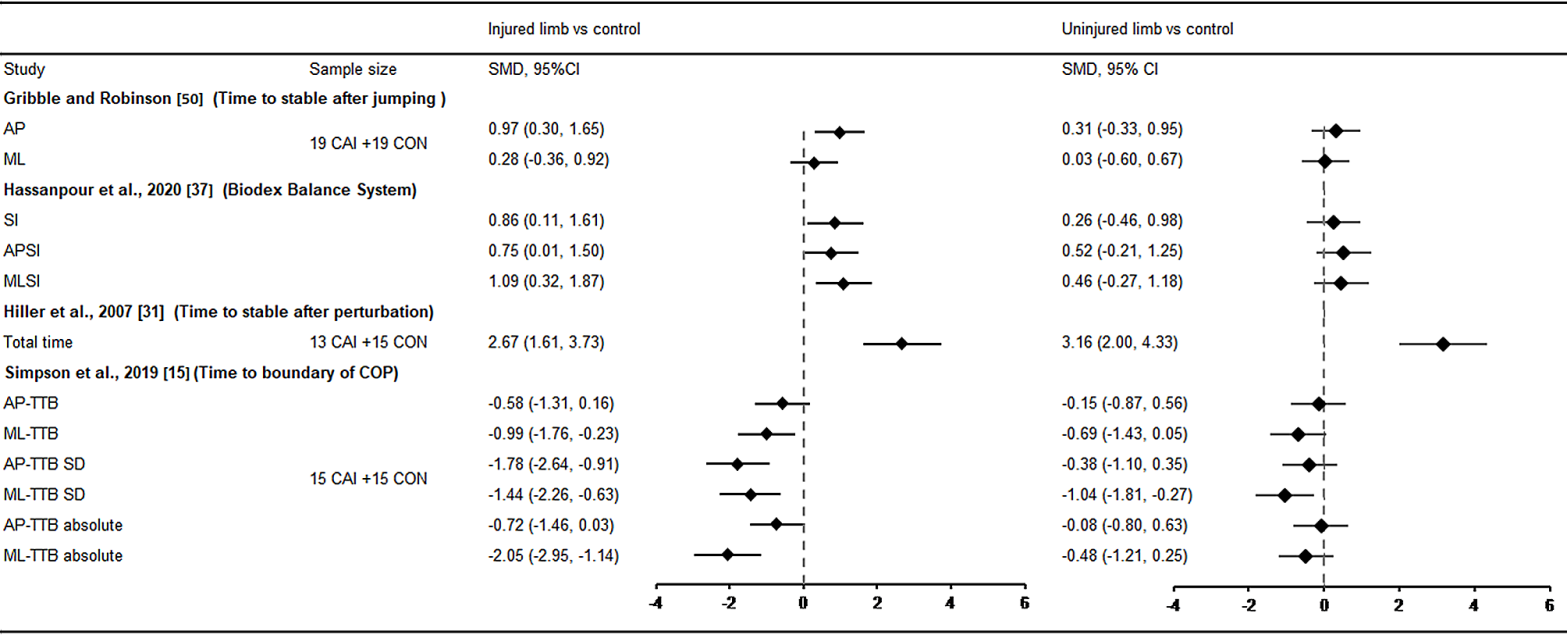
SMD values and 95% CI for dynamic balance tests. AP anteroposterior, APSI anteroposterior stability index, AP-TTB anteroposterior time to boundary, CAI chronic ankle instability, CI confidence interval, CON control, COP centre of pressure, ML mediolateral, MLSI mediolateral stability index, ML-TTB mediolateral time to boundary, SD standard deviation, SI stability index, SMD standardised mean difference. In the study by Simpson et al. [15], negative SMD indicates balance deficits in CAI. In other three studies [31, 37, 50], positive SMD indicates balance deficits in CAI

For dynamic balance, eight studies evaluated the normalised reach distance using the SEBT [32, 35–41], and data from these articles were analysed. The pooled results of SEBT revealed patients with CAI without bilateral deficits in any directions. Differences were only observed in the injured limb compared with the control in the anterior (SMD = − 0.34, 95% CI: − 0.60 to − 0.08, *I*^2^ = 0%), posteromedial (SMD = − 0.36, 95% CI: − 0.57 to − 0.14, *I*^2^ = 0%), anteromedial (SMD = − 0.41, 95% CI: − 0.73 to − 0.09, *I*^2^ = 0%), and medial (SMD = − 0.39, 95% CI: − 0.71 to − 0.07, *I*^2^ = 0%) directions (Table 1). In addition, Gribble et al. [50] investigated the time to maintain stability after jump landings and did not find evidence of bilateral deficits (Fig. 3). Another study [15] assessed dynamic balance using the lateral step-down test and observed that patients with unilateral CAI presented bilateral postural control impairments in mediolateral direction with large ES (SMD = − 1.44, 95% CI: − 2.26 to − 0.63 for injured limb vs. control; SMD = − 1.04, 95% CI: − 1.81 to − 0.27 for uninjured limb vs. control, Fig. 3). Hiller et al. [31] investigated the time to stabilise after perturbation and found bilateral instability with large ES (SMD = 2.67, 95% CI: 1.61 to 3.73 for injured limb vs. control; SMD = 3.16, 95% CI: 2.00 to 4.33 for uninjured limb vs. control, Fig. 3). Hassanpour et al. [37] applied the Biodex Balance System to evaluate bilateral balance in patients with CAI, revealing no evidence of bilateral deficits.

**Table 1:** Summary of pooled standardized mean differences (SMDs) with 95% confidence intervals (CIs) and overall heterogeneity for the Star Excursion Balance Test

Direction	Injured limb vs. control			Uninjured limb vs. control			Number of included studies
	SMD (95%CI)	Heterogeneity	*p*	SMD (95%CI)	Heterogeneity	*p*	
Anterior	−0.34(−0.60, −0.08)	I^2^ = 0%	0.011	−0.15(−0.42, 0.13)	I^2^ = 38%	0.298	8^[32, 35–41]^
Posterolateral	−0.18(−0.39, 0.03)	I^2^ = 0%	0.099	−0.12(−0.35, 0.11)	I^2^ = 12%	0.303	8^[32, 35–41]^
Posteromedial	−0.36(−0.57, −0.14)	I^2^ = 0%	0.001	−0.05(−0.26, 0.15)	I^2^ = 0%	0.610	8^[32, 35–41]^
Anteromedial	−0.41(−0.73, −0.09)	I^2^ = 0%	0.011	−0.08(−0.40, 0.23)	I^2^ = 0%	0.603	3^[37, 38, 41]^
Medial	−0.39(−0.71, −0.07)	I^2^ = 0%	0.016	−0.02(−0.40,0.37)	I^2^ = 28%	0.936	3^[37, 38, 41]^
Posterior	−0.27(−0.58, 0.05)	I^2^ = 0%	0.095	−0.28(−0.60, 0.03)	I^2^ = 0%	0.080	3^[37, 38, 41]^
Lateral	−0.25(−0.57, 0.06)	I^2^ = 0%	0.119	0.00(−0.31, 0.31)	I^2^ = 0%	1.000	3^[37, 38, 41]^
Anterolateral	−0.24(−0.56, 0.07)	I^2^ = 0%	0.131	−0.19(−0.51, 0.12)	I^2^ = 0%	0.234	3^[37, 38, 41]^

CI confidence interval, SMD standardised mean difference. A negative value of SMDs means that the CAI group (injured or uninjured limb) had deficits compared to the control. *p* refers to p-value of comparison

**Fig. 4:**
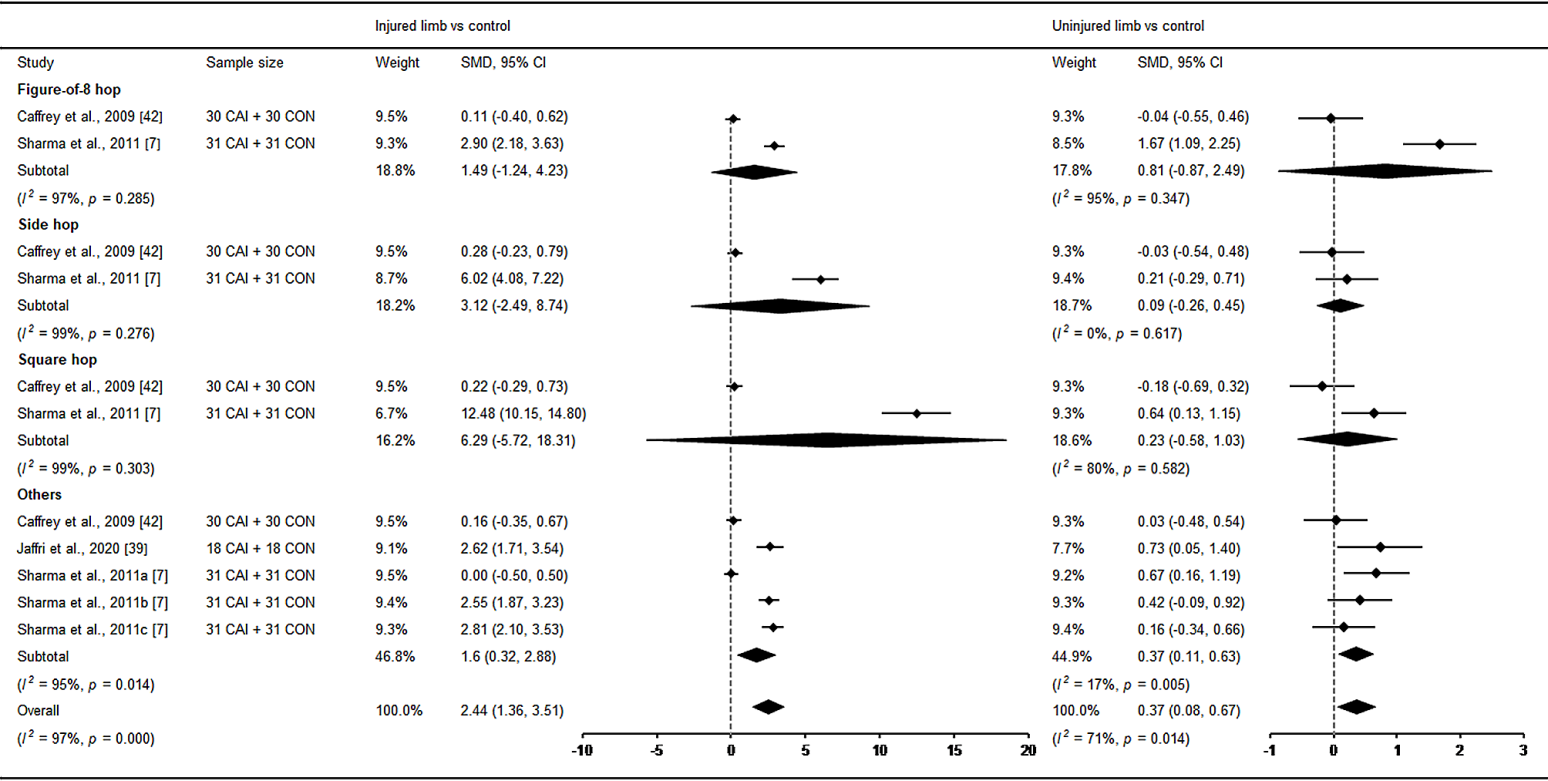
Forest plot of functional performance comparing CAI with control. Sharma et al., 2011a (single-limb hopping), b (Single-limb hurdle) and c (single hop) [7]. CAI chronic ankle instability, CON control, CI confidence interval, SMD standardised mean difference. Positive SMD indicates functional performance deficits in CAI

Functional performances were examined by three studies [7, 39, 42], with Caffrey et al. [42] and Sharma et al. [7] investigating multiple functional tests, including the figure-of-8 test, side hop, square hop and other kinds of hop tests (i.e., single-limb hopping or hurdle, 6-meter crossover hop and dynamic hop). As shown in Fig. 4, the pooled outcome showed a large deficit in the injured limb compared with control (SMD = 2.44, 95% CI: 1.36 to 3.51, *I*^*2*^ = 97%). The uninjured limb also displayed weak impairment compared with control (SMD = 0.37, 95%CI: 0.08 to 0.67, *I*^*2*^ = 71%). However, in the subgroup analysis, only the subgroup of ‘others’ presented significant differences bilaterally (SMD = 1.6, 95% CI: 0.32 to 2.88, *I*^*2*^ = 95% for injured limb vs. control; SMD = 0.37, 95% CI: 0.11 to 0.63, *I*^*2*^ = 17% for uninjured limb vs. control); the other subgroups all indicated no difference. The funnel plots of static and dynamic balance, as well as functional performance are displayed in Supplementary Material 5, indicating no publication bias in the meta-analysis comparing the uninjured limb with control.

### Muscle Strength and Activation

**Fig. 5:**
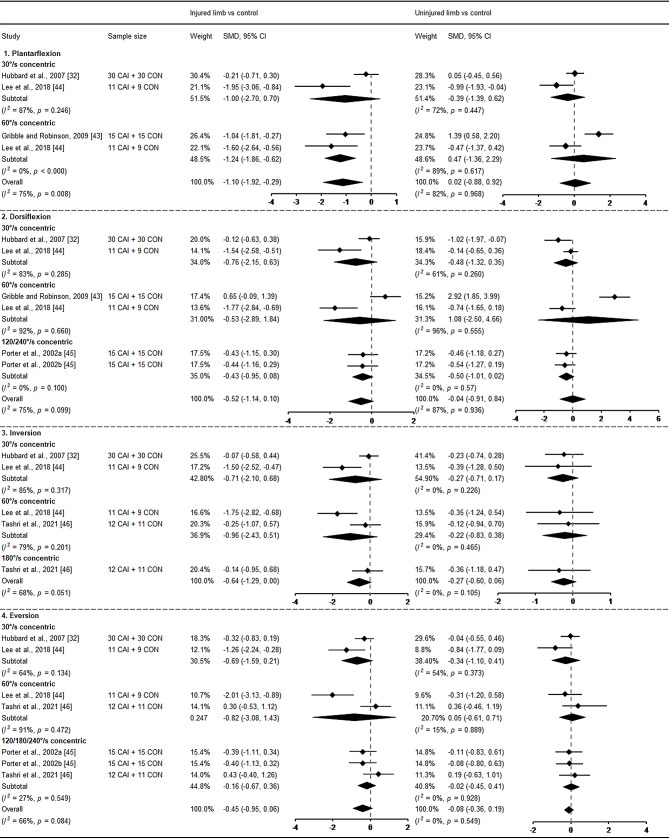
Forest plot of muscle strength comparing CAI with control. Porter et al., 2022a (120°/s concentric) and b (240°/s concentric) [45]. CAI chronic ankle instability, CON control, CI confidence interval, SMD standardised mean difference. Negative SMD indicates strength deficits in CAI

**Fig. 6:**
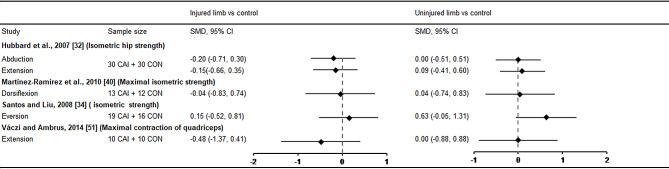
SMD values and 95% CI for isometric and maximal muscle contraction. CAI chronic ankle instability, CON control, CI confidence interval, SMD standardised mean difference. Negative SMD indicates strength deficits in CAI

Eight studies explored muscle strength at the ankle [32, 34, 40, 43–46, 51], specifically including dorsiflexor, plantarflexor, invertor and evertor. Among these, five studies investigated isokinetic concentric strength and pooled data for meta-analyses [32, 43–46]. In terms of plantarflexor, the injured side demonstrated significantly decreased muscle strength (SMD = − 1.01, 95%CI: −1.92 to − 0.29, *I*^*2*^ = 75%) compared with control with a large ES with 95% CI that did not cross zero, and no difference was found in dorsiflexor, evertor and invertor (Fig. 5). No significant difference was found in the uninjured limb compared with healthy controls, suggesting no bilateral deficits in ankle isokinetic concentric muscle strength in patients with unilateral CAI. In addition, Santos and Liu [34] conducted a study on bilateral isometric evertor strength in individuals with FAI and healthy controls. Martínez-Ramírez [40] focused on assessing bilateral maximal dorsiflexor strength, Váczi and Ambrus [51] investigated bilateral 60°/s and 180°/s peak concentric torque, 60°/s peak eccentric torque and maximal voluntary isometric torque at knee extensors. Hubbard et al. [32] assessed bilateral hip abductor and extensor strength in patients with CAI and healthy controls. None of these four studies found any bilateral deficits (Fig. 6).

**Fig. 7:**
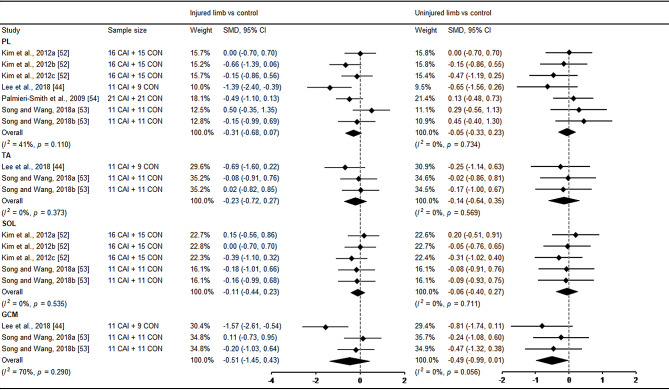
Forest plot of muscle activation magnitude comparing CAI with control. Kim et al., 2012a (prone), b(bipedal) and c(unipedal) [52]; Song and Wang, 2018a (before landing) and b (inversion perturbation after landing) [53]. CAI chronic ankle instability, CON control, CI confidence interval, SMD standardised mean difference. Negative SMD indicates activation deficits in CAI

**Fig. 8:**
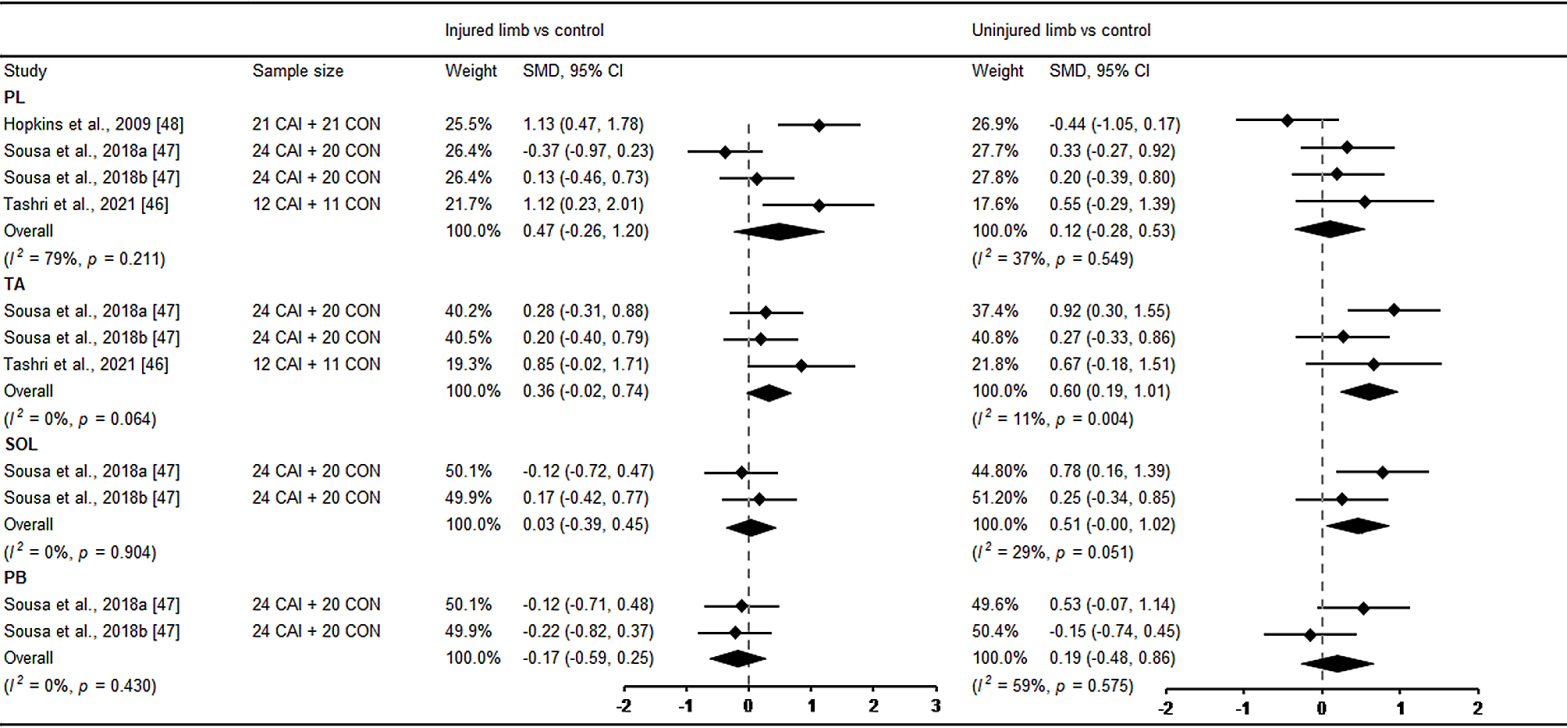
Forest plot of muscle activation time comparing CAI with control. Sousa et al., 2018a(support) and b(perturbation) [47]. CAI chronic ankle instability, CON control, CI confidence interval, SMD standardised mean difference. Positive SMD indicates delayed activation in CAI

For the results of muscle activation, six studies investigated muscle activation using sEMG and one study applied tensiomyography assessment during postural control in patients with CAI [44, 52–54]. Regarding activation magnitude, no significant differences were found in peroneus longus, tibialis anterior, soleus and gastrocnemius between CAI and healthy control (Fig. 7). In terms of activation time, only the uninjured limb revealed later activation in tibialis anterior compared with healthy control (Fig. 8).

### Sensation

**Fig. 9:**
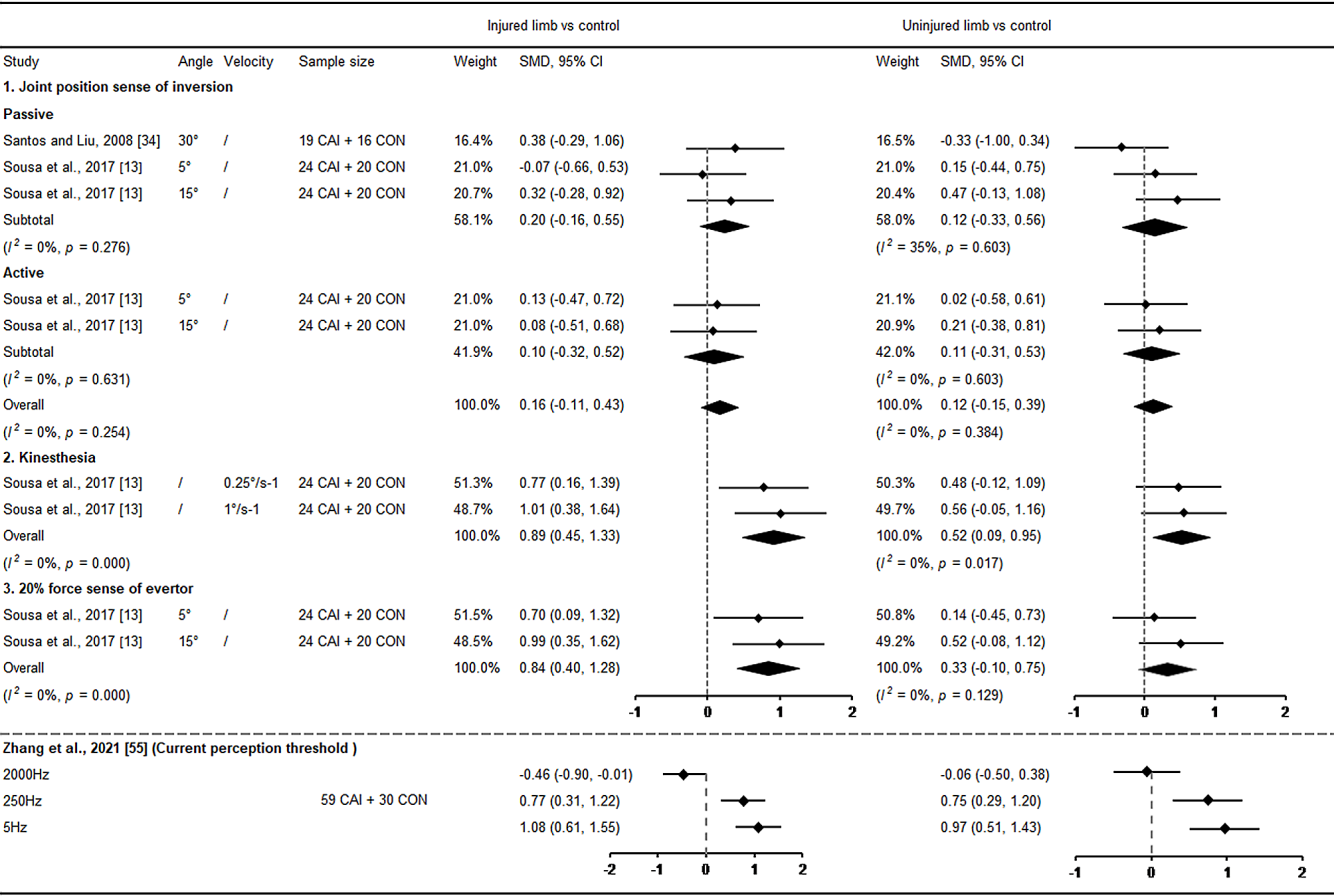
Forest plot of sensation at the ankle comparing CAI with control. CAI chronic ankle instability, CON control, CI confidence interval, SMD standardised mean difference. Positive SMD indicates sensory deficits in CAI

Only two studies investigated bilateral proprioception at the ankle (Fig. 9) [13, 34]. The meta-analysis revealed a large deficit in the injured side (SMD = 0.89, 95% CI: 0.45 to 1.33, *I*^*2*^ = 0%) and a moderate deficit in the uninjured (SMD = 0.52, 95% CI: 0.09 to 0.95, *I*^*2*^ = 0%) side compared with control in kinaesthesia. Furthermore, the injured limb showed a higher 20% force sense error in eversion (SMD = 0.84, 95% CI: 0.40 to 1.28, *I*^*2*^ = 0%) compared with control, while there was no significant difference in the uninjured limb. No significant differences were found between injured and uninjured limbs in active and passive inversion of joint position sense compared with control. In addition, Zhang et al. [55] reported high current perception threshold in both ankles of patients with unilateral CAI at frequencies of 250 Hz and 5 Hz compared with healthy controls, implying dysfunction in A-delta fibres transmitting fast pain signals and C fibres transmitting slow pain signals.

### Sensitivity Analysis

The sensitivity analysis showed that, in some cases, after excluding studies with large effects, the pooled results or effects were affected. In comparison of the ‘injured limb versus control’, after excluding the study by Sharma et al. [7] from the subgroup of ‘others’ in functional performance analysis or the study by Gribble et al. [43] from plantarflexor strength analysis did not yield significant differences. In addition, removing the study conducted by Sousa et al. [13], which investigated 1°/s^− 1^ kinesthesia and 20% evertor force sense, changed the effects from large to moderate. In comparison of the ‘uninjured limb versus control’, the pooled outcome of dorsiflexor presented a small effect after removal of the study by Gribble et al. [43].

## Discussion

This present study aimed to determine whether unilateral CAI presented bilateral sensorimotor deficits in balance, functional performance, muscle strength and activation and sensation. Taking the overview, the pooled results and evidence indicated the presence of sensorimotor deficits in both limbs of unilateral CAI in some specific aspects compared with healthy controls. Aside from the fact that CAI presented sensorimotor deficits in the injured side, in some cases, the uninjured side indicate static balance with eyes open and functional performance impairments with small ES, along with moderate kinaesthesia deficit and later tibialis anterior activation.

### Injured Limb Versus Control

The injured limb of patients with CAI showed decreased static and dynamic balance, constrained functional performance, reduced muscle strength and increased proprioception errors compared with healthy controls. These findings were consistent with previous studies [4, 6, 10–12, 17], but minor differences were observed. The meta-analysis of Song et al. [11] reported that the CAI had dynamic balance deficit in the anterior, posterolateral and posteromedial directions, but the current study indicated no deficit in the posterolateral direction. Furthermore, no difference was found in the subgroup analysis of ‘side hop’, which was contradictory to the previous meta-analysis [12]. No difference was observed in the active inversion joint position sense in the injured limb, which is contradicted by the results of Xue et al. [6] The meta-analyses of muscle strength were also not entirely consistent with the study by Khalaj et al. [4] These differences can mainly be attributed to the limited sample sizes in the comparisons of ‘injured limb versus control’. To achieve the objective of examining bilateral sensorimotor function in patients with unilateral CAI compared with healthy individuals, our study specifically included articles that evaluated bilateral sensory or motor function in unilateral CAI and healthy controls. Articles focusing solely on the injured side of CAI or those considering bilateral CAI cases were excluded. Consequently, our study may exhibit potential selection bias, distinguishing it from other meta-analyses that solely investigated function on the injured side of CAI, which resulted in minor distinctions in some comparisons between the injured side and healthy individuals. In addition, the heterogeneity or severity of patients with CAI included in this study may differ from that of other meta-analyses, resulting in dissimilar results.

### Uninjured Limb Versus Control

This comprehensive meta-analysis suggested that static balance with eyes open, functional performances, ankle kinaesthesia and later tibialis anterior activation deficits presented in the uninjured limb of patients with CAI.

For static balance with eyes open, bilateral instability presented in patients with unilateral CAI, but this result was contradictory with the previous meta-analysis that suggested the presence of bilateral deficits in acute lateral ankle instability rather than in CAI [17]. The inconsistency could be attributed to the different studies included and the different assessment methods for postural control. The studies included in the review conducted by Wikstrom et al. [17] were published before 2010 and summarised data mainly for static balance in the mediolateral direction with eyes open, while this current study assessed overall static balance with eyes open, potentially resulting in different outcomes. In addition, it should be noted that different parameters of assessment were employed. The study conducted by Wikstrom et al. [17] considered parameters including COP based parameters, sway index, and COP coordination when evaluating single-leg standing balance, while our study focused on the COP parameter which is the most commonly used parameter to evaluate static balance [56]. This difference in assessment methods may have contributed to the disparities observed in the pooled results of these two reviews. Interestingly, the pooled results showed no impairment in the uninjured limb compared with healthy controls in dynamic balance assessed by SEBT assessment and static stability with eyes closed. In comparison to static balance, dynamic balance and balance with eyes-closed requires higher ability of postural control. Therefore, the ambivalent results and small effects prevent us from drawing a strong conclusion.

However, it seems that sensorimotor impairments in uninjured limb could be assessed by more challenging tasks that require higher neuromuscular control ability. The pooled results of functional performance presented significant differences in uninjured limb. This result was supported by the findings of Jaffri et al. [39], in which functional tests could detect dynamic deficits but SEBT could not. In addition to the factors mentioned above, the absence of significant differences in the pooled results of the SEBT on the uninjured side can be attributed to the compensatory adjustment in the trunk and the early activation of the proximal muscles compensating for postural instability in lower limb [57]. Considering the practice trials before the SEBT test, the feedforward response during the formal test may allow individuals to adapt the potential deficit on the uninjured side [5].

**Fig. 10:**
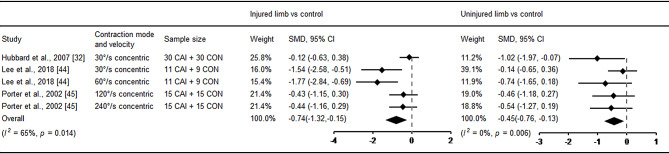
Sensitivity analysis for dorsiflexion. CAI chronic ankle instability, CON control, CI confidence interval, SMD standardised mean difference. Negative SMD indicates dorsiflexor strength deficit in CAI

In addition, pooled results suggested that CAI may present delayed muscle activation rather than reduced strength. High heterogeneity was observed in plantarflexor and dorsiflexor. The sensitivity analysis showed impaired dorsiflexor strength (SMD = − 0.45, 95% CI: −0.76 to − 0.13, *I*^*2*^ = 0%, Fig. 10) in uninjured limbs compared with healthy controls after the study by Gribble et al. [50] was removed. Interestingly, when the same study was excluded from the comparison of ‘injured limb versus control’, the outcome showed significance as well (SMD = − 0.74, 95% CI: −1.32 to − 0.15, *I*^*2*^ = 65%). For the plantarflexor, high heterogeneity was also caused by the study by Gribble et al. [50], and the heterogeneity changed from 82% to 49% after it was removed.

For sensation, impaired kinaesthesia was found in the uninjured side rather than joint position sense and force sense possibly because of the limited studies and small sample size, which may have caused inconsistent findings. Furthermore, in terms of neurophysiology, the muscle spindle is the main source of kinaesthetic information with position and movement [58–61], and it consists of two endings [58, 61]. The primary endings respond to alter the length and velocity of the muscle, and sense of position and movement, while the secondary endings carry length information and position sense alone [58, 61]. The tendon organs are located at the end of the muscle fibre and monitor the muscle tension, and they project afferents to the cerebral cortex [61]. Considering that the reorganisation of central pathways after ankle sprains may affect the primary endings, it leads to sense of movement discrepancy. Although the cutaneous and joint receptors are available for movement sense, they cannot compensate for the major loss from the muscle spindle.

These findings suggested that constrained sensorimotor functions in the injured ankle may affect the contralateral limb. Several mechanisms may explain the bilateral changes in the sensorimotor system of CAI. First, it was noticed that unilateral training leads to improving functions in bilateral limbs [62]. Hale et al. [63] revealed unilateral balance training in the uninjured side of CAI could enhance bilateral balance, providing further support for this mechanism. This mechanism called ‘cross education’ suggests that the efficacy of neural elements in the opposite limb could be influenced by high-force, unilateral, voluntary contractions through exercise, electrical stimulation or motor imagery [62]. Long-lasting training could activate neural circuits that chronically modify the motor pathways in the opposite limb and lead to functional improvement [62]. In contrast, the impairment in one side might affect the contralateral side. ‘Cross education’ seems to involve spinal and supraspinal modulation. It is believed that the involvement of relevant cortical mechanisms entails an intricate network consisting of inter-hemispheric connections and ipsilateral corticospinal fibers originating from the primary motor cortex, and these neural pathways play a crucial role in generating neural impulses that drive the contralateral muscle during unilateral movements [64, 65]. Activation of contralateral motor areas is similar in both sides demonstrated by functional magnetic resonance imaging during unilateral activation, implying inter-hemispheric interactions [66].

Another possible explanation is the coupled neural circuits that control both limbs. Edgley et al. [67] reported a group of interneurons that received bilateral joint afferents. From this perspective, the peripheral input conveyed by these afferent pathways may play a pivotal role in coordinating movements between limbs. This could elucidate the adverse impact observed when there is unilateral impairment affecting the postural-control responses of the contralateral limb.

In addition, the γ motoneuron activation is modulated by articular afferents, muscle, cutaneous and supraspinal commands [5]. Muscle spindle with high γ motoneuron activation will improve the feedback and feedforward control to modulate posture [68]. However, ankle sprains may damage mechanoreceptors at the ankle complex, including muscle spindles, Golgi tendon organs, Ruffini endings and Pacinian corpuscles [69], resulting in decreased or inaccurate sensory afferents on the injured side. Thus, the reduction in ankle-complex afferents could contribute to suppressed γ motoneuron activation [5]. Although integration of sensory information received from all parts of the body is largely considered to begin at the level of the spinal cord, some sensory information directly travels to the cortex without synapsing [5]. The decreased sensory input contributes to integration of spinal and supraspinal levels, leading to reorganisation or adaptive alterations and decreased motor output [19]. Although corticospinal fibres from the primary motor cortex predominantly control contralateral movements, a small number of fibres travel down to the ipsilateral side and control ipsilateral movements, which may influence motor functions in patients with CAI bilaterally [18]. In addition, Zhang et al. [55] found higher pain threshold in both ankles in unilateral CAI. Based on the theory of diffuse noxious inhibitory control, following heterotopic noxious stimulation, there is an inhibitory modulation of pain pathways. This modulation involves the subnucleus reticularis dorsalis and its descending projections to wide-dynamic-range neurons. In short, reduced sensory input may result in adaptation, reorganisation or inhibition in the central nervous system, and these changes may potentially influence the bilateral functions in unilateral CAI.

Finally, the bilateral sensorimotor features were different from the healthy controls in those individuals before the onset of CAI, and the sensorimotor differences could even contribute to the condition’s development [16]. However, this hypothesis is still unclear because of the lack of longitudinal studies.

### Research Implications

First, this current meta-analysis indicated the presence of bilateral sensorimotor deficits in unilateral CAI partially. Future research should try to assess both lower limbs’ function in patients with unilateral CAI to provide more evidence. Second, although appropriate explanations and hypotheses were made as to why bilateral sensorimotor deficits appear in CAI, the mechanisms still require further studies to investigate. Finally, owing to the different etiology and clinical manifestation of FAI and MAI, the current findings are less applicable, and further investigations are needed to determine whether bilateral impairments are present between the different types of CAI. For dynamic balance, SEBT appears to be deficient in detecting bilateral differences between CAI and healthy controls, and more precise methods or challenging postural tasks are needed.

### Clinical Implications

The findings of this meta-analysis and systematic review highlight the importance of assessing bilateral functions in patients with unilateral CAI. The novel consideration of our findings is that it provided a quantitative summary of the presence of sensorimotor deficits in the injured and uninjured side of CAI compared with healthy control in some specific aspects, especially balance and sensation. It also has a potential implication that the injured limb may cause the uninjured limb to develop CAI. Therefore, bilateral assessment is vital for sports training and rehabilitation in individuals with CAI, and bilateral training especially targeting balance and proprioception may also become important after injuries.

Furthermore, based on learning and neuroplasticity mechanisms, training the uninjured side may improve bilateral functions in patients with CAI [63]. Considering that the uninjured side owns intact sensory input structures, the neuroplasticity may be faster and more effective during neuromuscular retraining than the injured side. Hence, implementing interventions that specifically target the uninjured side of the ankle during the initial injury phase or in the early stages of CAI may expedite the improvement of sensorimotor function bilaterally. This approach holds the potential to enhance an athlete’s prospects of returning to play promptly and reduce the duration of the rehabilitation intervention process.

### Study Limitations

Some limitations must be considered when interpreting the results. Firstly, the high heterogeneity of many of the meta-analyses limits the ability to draw strong conclusions and suggests many aspects should be viewed with caution. In addition, for proprioception, related studies are few, thus reducing the reliability of the pooled results. For meta-analyses of static balance and functional performance on the injured side, the pooled ES was small, and more large-sample studies are needed to explore the bilateral functions in CAI.

## Conclusions

The findings of this systematic review and meta-analysis suggested that bilateral sensorimotor deficits, including static balance with eyes open, functional performance and kinaesthesia may present in unilateral CAI. However, due to the limited studies and some high heterogeneity in some pooled results, more related evidence is required to confirm this finding. Future studies should further investigate bilateral functions of unilateral CAI to provide useful information about potential mechanisms and rehabilitation methods.

### Electronic Supplementary Material

Below is the link to the electronic supplementary material.

Supplementary Material 1

Supplementary Material 2

Supplementary Material 3

Supplementary Material 4

Supplementary Material 5

## Data Availability

Not applicable.

## Abbreviations

CAI: chronic ankle instability
CIs: confidence intervals
COP: centre of pressure
EAI: Epidemiological Appraisal Instrument
FAI: functional ankle instability
MAI: mechanical ankle instability
SEBT: Star Excursion Balance Test
sEMG: surface electromyograph
SMD: Standardized mean diference
