# Supplementary material for: Bilateral Sensorimotor Impairments in Individuals with Unilateral Chronic Ankle Instability: A Systematic Review and Meta-Analysis
Source: Sports Med Open. 2024 Apr 8;10:33. doi: 10.1186/s40798-024-00702-y (PMC11001848; doi:10.1186/s40798-024-00702-y)
Supplement: Supplementary file 1 — Supplementary Material 1 [file 40798_2024_702_MOESM1_ESM.docx]

**Supplementary 1. Study characteristics**

| **Author, year** | **Study design** | **Background of participants** | **N of Patients** | **Age of Patients** | **Gender of patients** | **N of control** | **Age of control** | **Gender of control** | **Outcome measures** | **Methods** | **Measurement** | **Related outcomes** |
| --- | --- | --- | --- | --- | --- | --- | --- | --- | --- | --- | --- | --- |
| Caffrey,2009 | cross-sectional | university students | 30 | 20.5±1.6 | 15M/15F | 30 | 20.0±1.0 | 15M/15F | functional performance | figure-of-8 hop, side hop, 6-meter crossover hop, square hop | time to complete tests | Significantly longer time to complete tests on the injured side compared with the uninjured side and control. |
| Doherty,2016 | case-control | hospital patients | 28 | 23.2±4.13 | 17M/11F | 20 | 22.5±1.6 | 15M/5F | dynamic balance | Y balance | normalized reach distance | Significant lower values in CAI bilaterally compared with control in three directions. |
| Fusco, 2019 | cross-sectional | _ | 15 | 23.6±1.7 | _ | 15 | 22.6±1.3 | _ | dynamic balance | Y balance | normalized reach distance | Significant bilaterally higher value in CAI on the anterior direction. |
| Gribble,2009 | case-control | university students | 15 | 20.3±2.9 | 7M/8F | 15 | 23.1±3.9 | 7M/8F | muscle strength | isokinetic dynamometer | peak torque | Significant lower plantarflexion, knee flexion, and knee extension strength in the injured side compared with the uninjured side and control. |
| Hadadi,2011 | cross-sectional | university students | 20 | 23.6±3.6 | 12M/8F | 20 | 23.1±3.3 | 12M/8F | static balance | force platform | ML and AP COPV | Significant higher COPV in both sides of CAI. |
| Hassanpour,2020 | cross-sectional | university students | 15 | 25±5.32 | _ | 15 | 23.7±4.69 | _ | dynamic balance | SEBT, Biodex Balance System | normalized reach distance | Significantly lower reach distance in the injured side of CAI compared with the control. |
| Hertel，2006 | case-control | young adults | 48 | 20.9 ± 3.2 | 22M/26F | 39 | 20.7 ± 2.4 | 23M/16F | dynamic balance | SEBT | normalized reach distance | Significant lower value in the injured side of CAI compared with the uninjured side and control except for the medial direction. |
| Hertel，2007 | case-control | physically active females | 15 | 19.7±1.3 | 15F | 9 | 22.7 ±2.6 | 9F | static balance | accusway force platform | ML and AP COPV, TTB | Significantly lower TTB values in the injured side compared with the control. |
| Hiller, 2007 | cross-sectional | university and general community individuals | 19 | 23.7 ±6.6 | _ | 20 | 24.5±9.9 | _ | static balance | the three-dimensional electromagnetic tracking device | COP sway | No significant difference between and within the group. |
| Hubbard, 2007 | case-control | _ | 30 | 20.3 ± 1.3 | 15M/15F | 30 | 21.3±3.8 | 15M/15F | static and dynamic balance, muscle strength | accusway force plate, Y balance, Biodex Balance System, isokinetic dynamometer | COPV, normalized reach distance, 30°/s concentric contraction | A significant difference between groups and limbs in plantarflexion and Y balance. |
| Jaffri, 2019 | case-control | _ | 18 | 20.83±1.29 | 10M/8F | 18 | 20.83±1.38 | 10M/8F | functional performance, dynamic balance | dynamic leap balance test, Y balance | time to complete test, normalized reach distance | Significant higher value in the injured side compared with the uninjured side and control. |
| Lee, 2018 | cross-sectional | university students | 11 | 21.72±0.78 | 5M/6F | 9 | 22.33±1.58 | 4M/5F | isokinetic strength, muscle activity | isokinetic dynamometer, sEMG | 30°/s and 60°/s concentric contraction | Significant lower plantarflexion, dorsiflexion, inversion, and eversion of 60°/s in the injured side compared with the uninjured side and control. sEMG of gastrocnemius and peroneus longus were significantly decreased on the injured side compared with the uninjured side and control. |
| Martínez-Ramírez ,2010 | case-control | _ | 13 | 24.69 ±5.91 | 6M/7F | 12 | 23.16 ±5.32 | 7M/5F | dynamic balance | SEBT | normalized reach distance | No significant difference between and within groups. |
| Mitchell, 2008 | case-control | university students | 19 | 26.5±3.1 | 19M | 19 | 25.1 ±3.9 | 19M | static balance | Kistler force platform | COP sway | Significantly greater COP sway in the injured side with eyes open compared with the uninjured side and control. |
| Olmsted, 2002 | case-control | university students | 20 | 19.8±1.4 | 10M/10F | 20 | 20.2±1.4 | 10M/10F | dynamic balance | SEBT | normalized reached distance | Significant lower values in the injured side compared with the uninjured side and control. |
| Porter, 2002 | case-control | university students | 15 | 22.1 ± 3.7 | 6M/9F | 15 | 21 ±3.1 | 6M/9F | muscle strength | isokinetic dynamometer | 120°/s and 240°/s concentric contraction | No significant difference between and within groups. |
| Santos, 2008 | case-control | medical center and university patents | 21 | 30 ±11 | 6M/15F | 16 | 31±11 | 4M/12F | static balance, muscle strength | force platform, isokinetic dynamometer | COP sway, 120°/s concentric contraction | Significantly greater COP sway in the injured side compared with the uninjured side. |
| Sharma, 2011 | case-control | athletes | 31 | 21.7±1.9 | 26M/5F | 31 | 21.6±1.8 | 26M/5F | functional performance | single-limb hopping, figure of-8-hop, side hop, single-limb hurdle, square hop, single hop | time to complete the test | Significant differences were observed for all tests except the single hop test between the injured side and uninjured side, control. |
| Sousa, 2017 | case-control | university student-athletes | 24 | 20.6±2.52 | 18M/6F | 20 | 21.8±2.21 | 17M/3F | Proprioception | joint position sense, kinesthesia, force sense | 5° and 15° inversion, 0.25°/s, 1°/s ,20% maximal isometric contraction | Significantly higher error in the injured side for eversion movement detection (kinesthesia) and evertor force sense and higher error in the uninjured limb for evertor force sense. |
| Tashri, 2021 | cross-sectional | physically active university students | 12 | 21.4 ± 1.4 | 11M/1F | 11 | 22.4 ± 1.4 | 9M/2F | muscle strength | isokinetic dynamometer | 60°/s and 180°/s concentric contraction | No significant muscle strength between and within groups. |

AP: anteroposterior, CAI: chronic ankle instability, COPV: velocity of center of pressure, F: female, M: male, ML: mediolateral, SEBT: star excursion balance test, sEMG: surface electromyography, TTB: time to the boundary.
