# Supplementary material for: Bilateral Sensorimotor Impairments in Individuals with Unilateral Chronic Ankle Instability: A Systematic Review and Meta-Analysis
Source: Sports Med Open. 2024 Apr 8;10:33. doi: 10.1186/s40798-024-00702-y (PMC11001848; doi:10.1186/s40798-024-00702-y)
Supplement: Supplementary file 2 — Supplementary Material 2 [file 40798_2024_702_MOESM2_ESM.docx]

**Supplementary 2. Quality assessment of included studies in meta-analysis.**

| **Author, year** | **Hypothesis/aim/objective clearly described.** | **Exposure variables clearly described.** | **Main outcomes clearly described.** | **Study design clearly described.** | **Source of subject population clearly described.** | **Eligibility criteria for subject clearly described.** | **Participation rate reported, ascertainment of record availability described** | **Characteristics of study participants described.** | **Characteristics of subjects lost after entry/subjects not participating. described.** | **Important covariates and confounders described.** | **Statistical methods clearly described.** | **Main findings of the study clearly described.** | **Study provide estimates of random variability in data for main outcomes.** | **Study provide estimates of the statistical parameters.** | **Sample size calculations performed and reported.** | **Comparison/reference group comparable to the exposed group.** | **Participation rate and ascertainment of record availability adequate.** | **Study subjects from different groups recruited over same period of time.** | **Subject losses or unavailable records were taken account.** | **Diagnosis of ankle instability reliable.** | **Diagnosis of ankle instability valid.** | **Methods of assessing the exposure variables similar for each group.** | **Observers blinded to subject groupings or disease status.** | **Main outcome measures reliable.** | **Main outcome measures valid.** | **Methods of assessing the outcome variables standard across all groups.** | **Observation taken over the same time for all groups.** | **Prior history of disease and/or injury collected and included in the analysis.** | **Adequate adjustment for covariates and confounders** | **Outcome data reported by levels of exposure.** | **Outcome/exposure data reported by subgroups of subjects.** | **Study results be applied to the eligible population.** | **Study results be applied to other relevant populations.** | **Overall EAI score (0~1)** |
| --- | --- | --- | --- | --- | --- | --- | --- | --- | --- | --- | --- | --- | --- | --- | --- | --- | --- | --- | --- | --- | --- | --- | --- | --- | --- | --- | --- | --- | --- | --- | --- | --- | --- | --- |
| Caffrey,2009 |  |  |  |  |  |  |  |  |  |  |  |  |  |  |  |  |  |  |  |  |  |  |  |  |  |  |  |  |  |  |  |  |  | 0.62 |
| Doherty,2016 |  |  |  |  |  |  |  |  |  |  |  |  |  |  |  |  |  |  |  |  |  |  |  |  |  |  |  |  |  |  |  |  |  | 0.53 |
| Fusco, 2019 |  |  |  |  |  |  |  |  |  |  |  |  |  |  |  |  |  |  |  |  |  |  |  |  |  |  |  |  |  |  |  |  |  | 0.45 |
| Gribble ,2009 |  |  |  |  |  |  |  |  |  |  |  |  |  |  |  |  |  |  |  |  |  |  |  |  |  |  |  |  |  |  |  |  |  | 0.56 |
| Hadadi,2011 |  |  |  |  |  |  |  |  |  |  |  |  |  |  |  |  |  |  |  |  |  |  |  |  |  |  |  |  |  |  |  |  |  | 0.50 |
| Hassanpour,2020 |  |  |  |  |  |  |  |  |  |  |  |  |  |  |  |  |  |  |  |  |  |  |  |  |  |  |  |  |  |  |  |  |  | 0.53 |
| Hertel, 2006 |  |  |  |  |  |  |  |  |  |  |  |  |  |  |  |  |  |  |  |  |  |  |  |  |  |  |  |  |  |  |  |  |  | 0.52 |
| Hertel，2007 |  |  |  |  |  |  |  |  |  |  |  |  |  |  |  |  |  |  |  |  |  |  |  |  |  |  |  |  |  |  |  |  |  | 0.44 |
| Hiller, 2007 |  |  |  |  |  |  |  |  |  |  |  |  |  |  |  |  |  |  |  |  |  |  |  |  |  |  |  |  |  |  |  |  |  | 0.62 |
| Hubbard, 2007 |  |  |  |  |  |  |  |  |  |  |  |  |  |  |  |  |  |  |  |  |  |  |  |  |  |  |  |  |  |  |  |  |  | 0.48 |
| Jaffri, 2019 |  |  |  |  |  |  |  |  |  |  |  |  |  |  |  |  |  |  |  |  |  |  |  |  |  |  |  |  |  |  |  |  |  | 0.55 |
| Lee, 2018 |  |  |  |  |  |  |  |  |  |  |  |  |  |  |  |  |  |  |  |  |  |  |  |  |  |  |  |  |  |  |  |  |  | 0.55 |
| Martínez-Ramírez ,2010 |  |  |  |  |  |  |  |  |  |  |  |  |  |  |  |  |  |  |  |  |  |  |  |  |  |  |  |  |  |  |  |  |  | 0.44 |
| Mitchell, 2008 |  |  |  |  |  |  |  |  |  |  |  |  |  |  |  |  |  |  |  |  |  |  |  |  |  |  |  |  |  |  |  |  |  | 0.44 |
| Olmsted,2002 |  |  |  |  |  |  |  |  |  |  |  |  |  |  |  |  |  |  |  |  |  |  |  |  |  |  |  |  |  |  |  |  |  | 0.52 |
| Porter, 2002 |  |  |  |  |  |  |  |  |  |  |  |  |  |  |  |  |  |  |  |  |  |  |  |  |  |  |  |  |  |  |  |  |  | 0.50 |
| Santos, 2008 |  |  |  |  |  |  |  |  |  |  |  |  |  |  |  |  |  |  |  |  |  |  |  |  |  |  |  |  |  |  |  |  |  | 0.56 |
| Sharma, 2011 |  |  |  |  |  |  |  |  |  |  |  |  |  |  |  |  |  |  |  |  |  |  |  |  |  |  |  |  |  |  |  |  |  | 0.52 |
| Sousa, 2017 |  |  |  |  |  |  |  |  |  |  |  |  |  |  |  |  |  |  |  |  |  |  |  |  |  |  |  |  |  |  |  |  |  | 0.55 |
| Tashri, 2021 |  |  |  |  |  |  |  |  |  |  |  |  |  |  |  |  |  |  |  |  |  |  |  |  |  |  |  |  |  |  |  |  |  | 0.52 |

Black = Reported, Grey = Partially reported, White = Not reported or Unable to determine.
