# Supplementary material for: Bilateral Sensorimotor Impairments in Individuals with Unilateral Chronic Ankle Instability: A Systematic Review and Meta-Analysis
Source: Sports Med Open. 2024 Apr 8;10:33. doi: 10.1186/s40798-024-00702-y (PMC11001848; doi:10.1186/s40798-024-00702-y)
Supplement: Supplementary file 3 — Supplementary Material 3 [file 40798_2024_702_MOESM3_ESM.docx]

**Supplementary 3. Risk of bias assessment of included studies in meta-analysis.**

| Author, year | External validity | Internal validity | | | | | |
| --- | --- | --- | --- | --- | --- | --- | --- |
|  | Performance | Detection | Attrition | Selection bias/control of confounding | | | |
|  | 1 | 2 | 3 | 4 | 5 | 6 | 7 |
| Caffrey,2009 |  |  |  |  |  |  |  |
| Doherty,2016 |  |  |  |  |  |  |  |
| Fusco, 2019 |  |  |  |  |  |  |  |
| Gribble ,2009 |  |  |  |  |  |  |  |
| Hadadi,2011 |  |  |  |  |  |  |  |
| Hassanpour,2020 |  |  |  |  |  |  |  |
| Hertel，2006 |  |  |  |  |  |  |  |
| Hertel，2007 |  |  |  |  |  |  |  |
| Hiller, 2007 |  |  |  |  |  |  |  |
| Hubbard, 2007 |  |  |  |  |  |  |  |
| Jaffri, 2019 |  |  |  |  |  |  |  |
| Lee, 2018 |  |  |  |  |  |  |  |
| Martínez-Ramírez ,2010 |  |  |  |  |  |  |  |
| Mitchell, 2008 |  |  |  |  |  |  |  |
| Olmsted,2002 |  |  |  |  |  |  |  |
| Porter, 2002 |  |  |  |  |  |  |  |
| Santos, 2008 |  |  |  |  |  |  |  |
| Sharma, 2011 |  |  |  |  |  |  |  |
| Sousa, 2017 |  |  |  |  |  |  |  |
| Tashri, 2021 |  |  |  |  |  |  |  |

Black: the measure was addressed adequately

White: the measure was not address adequately or clearly

1. Performance (Representativeness): if the study described demographic details, and were representative of “chronic ankle instability” population.

2. Detection (Blinded assessor): if data assessed or processed by a blinded assessor.

3. Attrition (Participation rate): if more than 80% of those who volunteered for the study were used eventually.

4. Appropriate description of characteristics of study participants: if they reported the character of chronic ankle instability (i.e. unilateral/bilateral or both, times of ankle sprain, or duration of symptoms) and control (i.e. no history of ankle sprain).

5. Appropriate testing protocol: if they used valid and reliable testing device.

6. Appropriate normalization: if between group comparisons normalized to a standard reference is described.

7. Appropriate statistical tests used: if describes type of tests.
