# Supplementary material for: Bilateral Sensorimotor Impairments in Individuals with Unilateral Chronic Ankle Instability: A Systematic Review and Meta-Analysis
Source: Sports Med Open. 2024 Apr 8;10:33. doi: 10.1186/s40798-024-00702-y (PMC11001848; doi:10.1186/s40798-024-00702-y)
Supplement: Supplementary file 4 — Supplementary Material 4 [file 40798_2024_702_MOESM4_ESM.docx]

**Supplementary 4. CAI Inclusion criteria of studies included in the systematic review based on the recommendation of the International Ankle Consortium.**

| Author, year | Population defined by authors | All criteria required | | | | At least 1 of 3 criteria required | | | Percentage of required criteria met |
| --- | --- | --- | --- | --- | --- | --- | --- | --- | --- |
|  |  | A history of at least 1 significant ankle sprain | Injury resulting in pain, swelling, interruption of physical activity for at least 1 day | The initial ankle sprain occurred at least 12 months prior to the study | No history of ankle sprain in last 3 months | At least 2 episodes of “giving way” in the last 6 months | Ankle sprain recurrence (at least 2 sprains on same ankle) | Self-reported ankle instability confirmed by questionnaires (AII, CAIT, IdFAI) |  |
| Caffrey,2009 | FAI |  |  |  |  |  |  |  | 0.5 |
| Doherty,2016 | CAI |  |  |  |  |  |  |  | 0.57 |
| Fusco, 2019 | CAI |  |  |  |  |  |  |  | 0.64 |
| Gribble ,2009 | CAI |  |  |  |  |  |  |  | 0.71 |
| Hadadi,2011 | FAI |  |  |  |  |  |  |  | 0.43 |
| Hassanpour,2020 | CAI |  |  |  |  |  |  |  | 0.43 |
| Hertel, 2006 | CAI |  |  |  |  |  |  |  | 0.64 |
| Hertel, 2007 | CAI |  |  |  |  |  |  |  | 0.57 |
| Hiller, 2007 | FAI |  |  |  |  |  |  |  | 0.43 |
| Hubbard, 2007 | CAI |  |  |  |  |  |  |  | 0.71 |
| Jaffri, 2019 | CAI |  |  |  |  |  |  |  | 0.86 |
| Lee, 2018 | FAI |  |  |  |  |  |  |  | 0.29 |
| Martínez-Ramírez ,2010 | CAI |  |  |  |  |  |  |  | 0.36 |
| Mitchell, 2008 | FAI |  |  |  |  |  |  |  | 0.64 |
| Olmsted,2002 | CAI |  |  |  |  |  |  |  | 0.29 |
| Porter, 2002 | FAI |  |  |  |  |  |  |  | 0.07 |
| Santos, 2008 | FAI |  |  |  |  |  |  |  | 0.5 |
| Sharma, 2011 | FAI |  |  |  |  |  |  |  | 0.64 |
| Sousa, 2017 | FAI, MAI |  |  |  |  |  |  |  | 0.86 |
| Tashri, 2021 | CAI |  |  |  |  |  |  |  | 0.71 |

Black = Reported, White = Not Reported, Grey = number or time frame not stated or not achieved the standard recommended.

CAI: chronic ankle instability; FAI: functional ankle instability; MAI: mechanical ankle instability.
