# Supplementary material for: Bilateral Sensorimotor Impairments in Individuals with Unilateral Chronic Ankle Instability: A Systematic Review and Meta-Analysis
Source: Sports Med Open. 2024 Apr 8;10:33. doi: 10.1186/s40798-024-00702-y (PMC11001848; doi:10.1186/s40798-024-00702-y)
Supplement: Supplementary file 5 — Supplementary Material 5 [file 40798_2024_702_MOESM5_ESM.docx]

Supplementary 5. Funnel plots for analyses of static balance with eyes open, dynamic balance and functional performance.


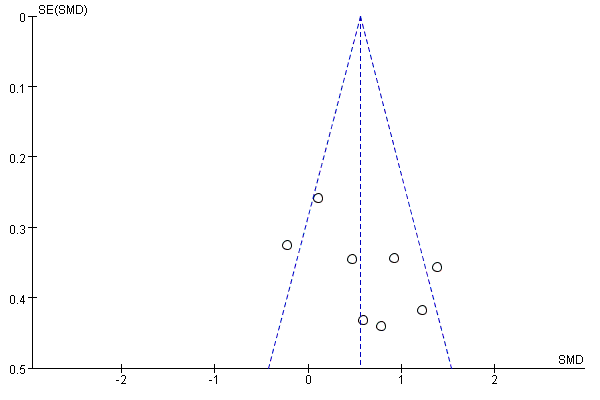


1. Injured limb vs. control for static balance with eyes open


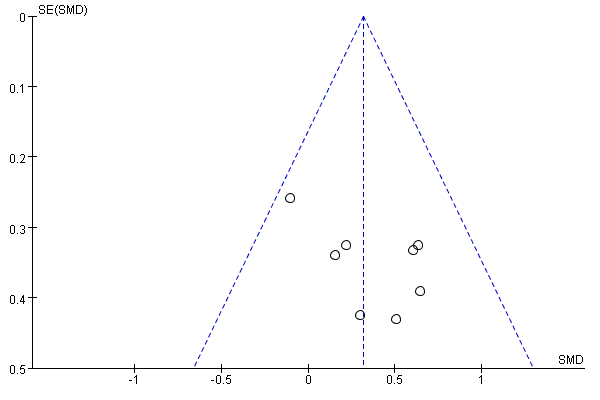


1. Uninjured limb vs. control for static balance with eyes open


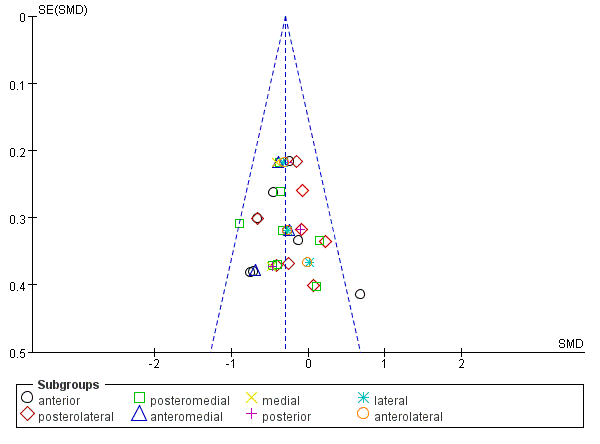


1. Injured limb vs. control for dynamic balance


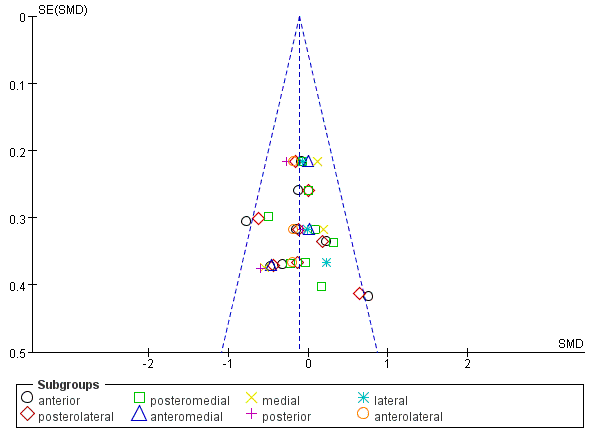


1. Uninjured limb vs. control for dynamic balance


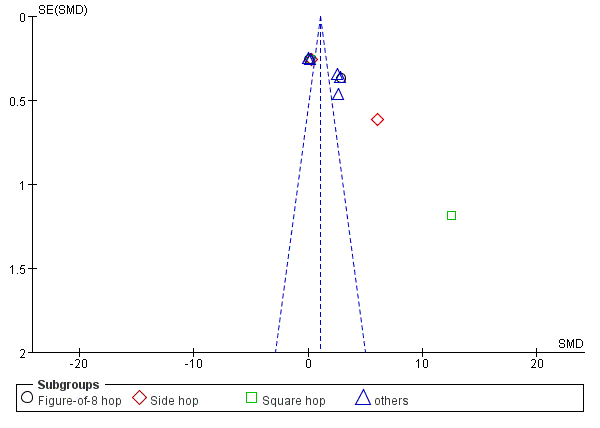


1. Injured limb vs. control for functional performance


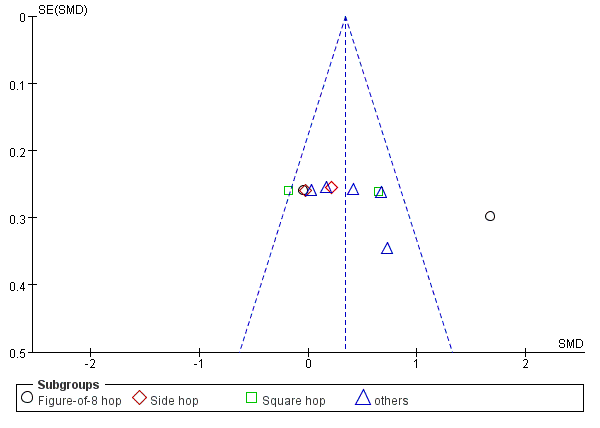


1. Uninjured limb vs. control for functional performance
